# Heart rate variability and pulmonary dysfunction in rats subjected to hemorrhagic shock

**DOI:** 10.1186/s12872-020-01606-x

**Published:** 2020-07-11

**Authors:** Fateme Khodadadi, Aminollah Bahaoddini, Alireza Tavassoli, Farzaneh Ketabchi

**Affiliations:** 1 Department of Biology, College of Sciences, Shiraz University, Shiraz, Iran; 2 Department of Pathology, Fasa University of Medical Sciences, Fasa, Iran; 3 Department of Physiology, School of Medicine, Shiraz University of Medical Sciences, Shiraz, Iran

**Keywords:** HRV, Lung injury, Shock, Vagus nerve

## Abstract

**Background:**

The activity of autonomic nervous system and its association with organ damage have not been entirely elucidated in hemorrhagic shock. The aim of this study was to investigate heart rate variability (HRV) and pulmonary gas exchange in hemorrhagic shock during unilateral subdiaphragmatic vagotomy.

**Methods:**

Male Sprague Dawley rats were randomly assigned into groups of Sham, vagotomized (Vag), hemorrhagic shock (HS) and Vag + HS. HS was induced in conscious animals by blood withdrawal until reaching to mean arterial blood pressure (MAP) of 40 ± 5 mmHg. Then, it was allowed to MAP returning toward the basal values. MAP and heart rate (HR) were recorded throughout the experiments, HRV components of low (LF, sympathetic index), high (LH, parasympathetic index), and very low (VLF, injury index) frequencies and the LF/HF ratio calculated, and the lung histological and blood gas parameters assessed.

**Results:**

In the initial phases of HS, the increase in HR with no change in MAP were observed in both HS and Vag + HS groups, while LF increased only in the HS group. In the second phase, HR and MAP decreased sharply in the HS group, whereas, only MAP decreased in the Vag + HS group. Meanwhile, LF and HF increased relative to their baselines in the HS and Vag + HS groups, even though the values were much pronounced in the HS group. In the third phase, HR, MAP, LF, HF, and the LF/HF ratio were returned back to their baselines in both HS and Vag + HS groups. In the Vag + HS group, the VLF was lower and HR was higher than those in the other groups. Furthermore, blood gas parameters and lung histology indicated the impairment of gas exchange in the Vag + HS group.

**Conclusions:**

The sympathetic activity is predominant in the first phase, whereas the parasympathetic activity is dominant in the second and third phases of hemorrhagic shock. There is an inverse relationship between the level of VLF and lung injury in vagotomized animals subjected to hemorrhagic shock.

## Background

Hemorrhagic shock is one of the leading causes of death in the world [1]. There are three phases of class II hemorrhagic shock, including the initial compensatory, decompensatory and recompensatory phases [2–5]. However, the mechanisms of different phases of compensation in hemorrhagic shock have not been fully elucidated thus far. It has been indicated that the compensatory responses to hemorrhagic shock depends on the integrity of afferent fibers of the vagus nerve originated from the arterial baroreceptors [2, 6, 7]. Besides, the subdiaphragmatic vagus nerves may be involved in regulating the autonomic nervous system’s negative feedback during different physiological conditions [8]. Nevertheless, little attention has been devoted about the role of subdiaphragmatic vagus nerve in the compensatory mechanisms of class II hemorrhagic shock yet. The fluctuation of RR interval duration is defined as heart rate variability (HRV). It has been expressed that the autonomic nervous system activity and the metabolic status of the body can be assessed by HRV [9, 10]. However, a few studies have addressed HRV in evaluating the autonomic activity in the early and compensatory phases of hemorrhagic shock [11, 12].

Inappropriate treatment of hemorrhagic shock may lead to body tissue damage and multi-organ failures. The lung is very vulnerable because it may be affected by different injurious substances carried by the blood through the pulmonary circulation from other injured tissues. In a few studies, the increase in pulmonary capillary permeability and the infiltration of immune cells into the pulmonary interstitial space have been shown in hemorrhagic shock [13]. On the other hand, the vagus nerve may play a role in regulating immune responses through anti-inflammatory cholinergic pathways [14]. However, the role of the subdiaphragmatic vagus nerve in lung injury induced by hemorrhagic shock remains unclear.

Based on the noted background, the present study aimed at investigating the alterations of hemodynamic and HRV at three phases of class II hemorrhagic shock in conscious rats. The relation between HRV, lung tissue damage and bloodborne parameters in three phases of hemorrhagic shock were also evaluated. Furthermore, the role of the subdiaphragmatic vagus nerve was assessed at the above conditions. This study was designed in conscious rats to exclude the effect of anesthetic drugs on the respiratory system-related heart rate variability.

## Methods

### Study design

This study was approved by the Medical Ethics Committee Regulations No IR.SUMS.MED.REC.1396.S203. Twenty four male Sprague-Dawley rats weighing 250–300 g were housed in standard cages under controlled laboratory temperature, humidity, and 12:12 h of light/dark cycles. They had free access to water and standard food and randomly assigned into four groups of Sham (*n* = 5), vagotomized (Vag, *n* = 5), hemorrhagic shock without (HS, *n* = 7) and with vagotomy (HS + Vag, *n* = 7). Animals were anesthetized by intraperitoneal injection of 50 mg/kg sodium pentobarbital (Sigma). Additional doses used as needed. The femoral vein was cannulated by a 120-PE catheter and fixed. The free end of the catheter was gently passed through the external part of the skin close to the base of the tail and fixed. Then, a 50 PE catheter was inserted into the tail artery and fixed firmly. Thereafter, the area of surgery was rinsed by 1% lidocaine (Sigma) to minimize the postoperative pain. Finally, The conscious animals were transferred to an optimized dark metabolic cage (MR Plexi), and the animals’ tails were fixed outside the cage so that they could relatively move in a cage without interrupting the hemodynamic recording. The arterial catheter was connected through a pressure transducer (MLT844) to a data acquisition system (Powerlab, PL26T04, ADinstruments, Australia). The mean arterial blood pressure (MAP) was recorded throughout the experiments and heart rate (HR) calculated accordingly. The femoral vein was used for blood sampling, and blood withdrawal during induction of hemorrhagic shock.

### Subdiaphragmatic vagotomy

After anesthesia, an incision was made in the upper abdominal skin. The fascia and muscles were dissected, and the left subdiaphragmatic vagus nerve, separated from the surrounding tissues, and cut apart. Then, muscles and skin were sutured, and the surgical areas were rinsed with 1% lidocaine. In the Sham group, animals underwent the arterial and venous cannulation and abdominal laparotomy, whereas, the vagus nerve remained intact.

### Study protocol: induction of hemorrhagic shock

**Fig. 1:**
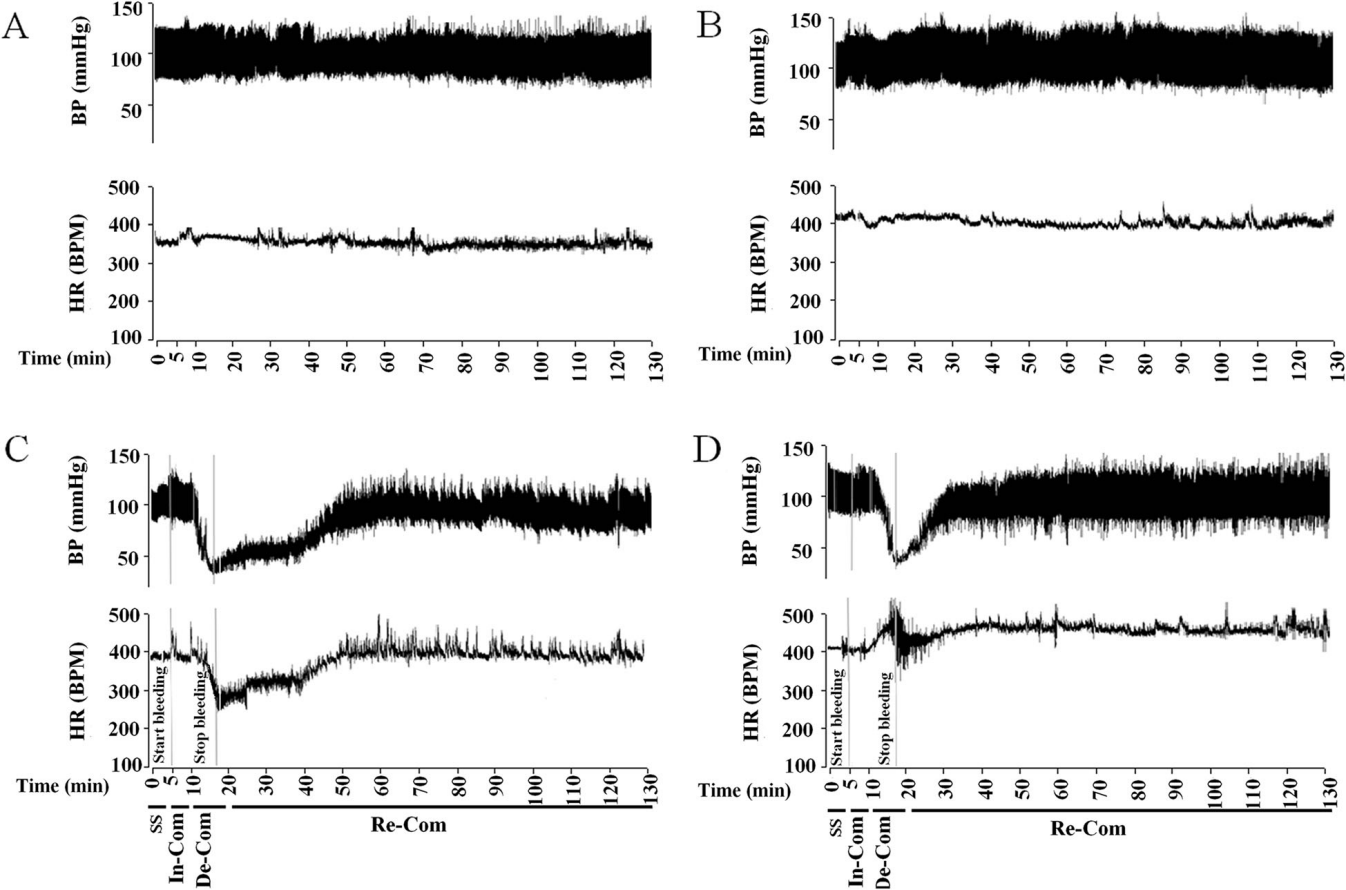
The real traces of blood pressure (BP) and heart rate (HR) recorded by a Powerlab system in the Sham (**a**), Vag (**b**), HS (**c**) and Vag + HS (**d**) groups. SS: Steady State; In-Com: initial Compensatory phase; De-Com: Decompensatory phase; Re-Com: Recompensatory phase

After vagotomy, and 70 min of steady-state period, the arterial and venous blood samples were taken for analyzing the blood gas parameters. Then, the HS and Vag + HS groups were subjected to hemorrhagic shock, according to the previous studies and our pilot experiments [12]. Briefly, the blood withdrawal with a constant flow rate of 0.5 ml/min/rat was taken from the femoral vein until the mean blood pressure reached to 40 ± 5 mmHg. The amount of blood withdrawal in the HS and Vag + HS groups were 26.07 ± 3.07%, and 23.37 ± 1.68% of body blood volume, respectively [15]. After that, the blood withdrawal was ceased so that it was allowed MAP returned to the pre-hemorrhage level without any resuscitation in 40 min based on the methods for class II hemorrhagic shock [16]. Two hours later, the arterial and venous blood samples were taken for blood gas analysis, hemoglobin concentration and plasma level of lactate. MAP and HR were recorded continuously throughout the experiments. Finally, animals were killed by high doses of pentobarbital and the lungs were prepared for histological analyses. All mentioned procedures were performed in the groups of Sham and Vag in the same time course as in the HS groups, except for the induction of hemorrhagic shock. Figure 1 demonstrates the real traces of blood pressure (BP) and heart rate (HR) in the experimental groups.

### HRV analysis

Kubios HRV premium/animal software (ver. 3.2) was used for HRV analysis. The interpolated techogram of 10 Hz [17], the Welch’s period diagram window width for 512 pieces and the hanning window with 50% overlap were selected. Based on previous studies, frequency-domain components of very low (VLF: 0 to 0.2 Hz), low (LF: 0.20 to 0.75 Hz), high-frequency (HF: 0.75 to 3.0 Hz) [18, 19], and LF/HF ratio were considered in this study. We used HRV analysis in three phases of class II hemorrhagic shock. The duration of all calculations were within 5 min of each consecutive 10 min. At each time interval, the pulse interval was calculated offline from the intervals of the peak systolic blood pressure. Interval pulse data were exported from the Kubios software to Excel. The HRV components of LF, HF, and VLF data were expressed as logarithm values (log).

### Arterial and venous blood gas parameters

The 200 μl of blood samples were taken during the baseline period and at the end of the experiments for the blood gas analyses using an easy blood gas device (Medica, USA). Total arterial and venous oxygen contents were calculated based on the following equation.

CaO_2_ = oxygen bound to Hb + oxygen in dissolved state = (1.34 × Hb × SaO_2_) + (PaO_2_ × 0.003); CvO_2_ = oxygen bound to Hb + oxygen in dissolved state = (1.34 × Hb × SvO_2_) + (PvO_2_ × 0.003).

### Histological evaluations

At the end of the experiments, the chest was opened and the lung removed and fixed in 4% formalin. Then, the samples were dehydrated by different concentrations of ethanol and xylol and embedded in paraffin. Tissue sections were prepared by a microtome (pfm medical, UK) and stained with hematoxylin and eosin [13]. All slides of histology were evaluated in a blinded manner by a pathologist. The pathological components of the focal thickening of the alveolar membranes, vascular congestion, perivascular neutrophil infiltration, and alveolar hemorrhage were evaluated. Each index was graded with a score of 0 to 3 based on the absence (0), mild (1), moderate (2) or severe (3) [13].

### Statistical analysis

Data are presented as means±SE. Analysis of variance (ANOVA) with Tukey’s post hoc test was used for comparisons of blood pressure and heart rate between groups. Also, repeated-measures ANOVA was used for comparison of data during the different time course of experiments. Furthermore, we compared non-parametric parameters including data of HRV and histology using Kruskal-Wallis and Mann-Whitney tests. We also used paired t-test for comparing blood variables at the onset and the end of the experiments. All analysis was performed using the software of SPSS 18. Significance was assumed when *P* < 0.05 and the confidence limits used were the 95% intervals.

## Results

**Fig. 2:**
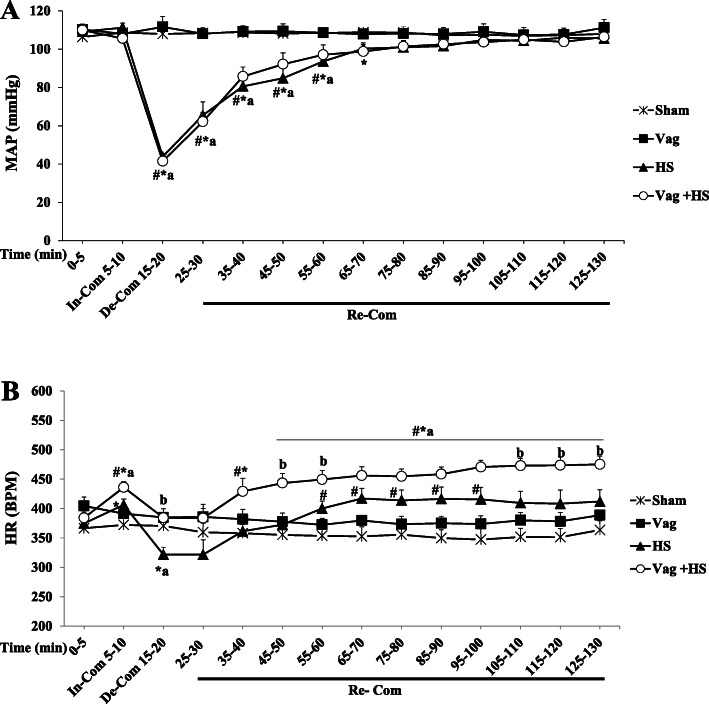
The Mean arterial blood pressure (MAP, **a**) and heart rate (HR, **b**) in the Sham (*n* = 5), Vag (*n* = 5), HS (*n* = 7) and Vag + HS (*n* = 7) groups. Data are mean ± SE. *P < 0.05 vs. the baseline; ^#^*p* < 0.05 vs. the Sham group; ^a^*p* < 0.05 vs. the Vag group; ^b^*p* < 0.05 vs. the HS group

MAP and HR in the Sham and Vag groups did not alter significantly throughout the experiments. During the In-Com phase of the HS group, MAP was maintained in the normal range, whereas HR increased significantly. The MAP then decreased, reaching to a minimum value of 43.8 ± 1.5 mmHg in the De-Com phase along with a sudden drop in HR. After cessation of blood withdrawal, MAP returned slowly during 40 min to its baseline level in the Re-Com phase. Also, HR in this phase was partially more than the ones in the Sham and Vag groups. During the In-Com and De-Com phases of the Vag + HS group, MAP were similar to those in the HS group. However, HR increased in the In-Com phase with no change in the De-Com phase. MAP in the Re-Com phase was similar to that in the HS group. Nevertheless, HR increased gradually being higher than those in the other groups in this phase (Fig. 2).

**Fig. 3:**
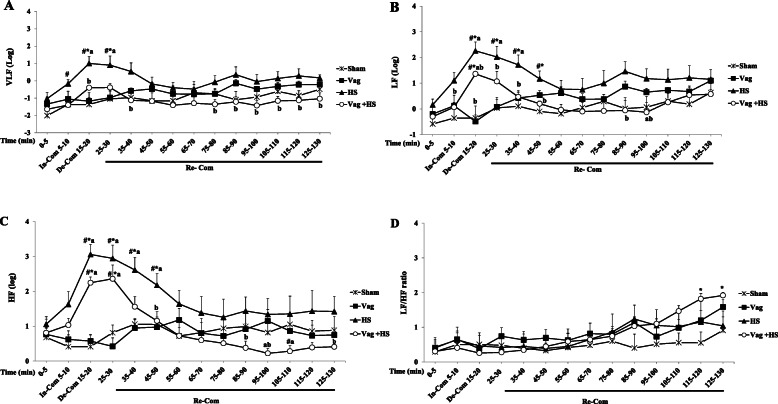
The frequency domain components of HRV including VLF (**a**), LF (**b**), HF (**c**) and LF/HF ratio (**d**) in the Sham (*n* = 5), Vag (*n* = 5), HS (*n* = 7) and Vag + HS (*n* = 7) groups. Data are mean ± SE. *P < 0.05 vs. the baseline; ^#^*p* < 0.05 vs. the Sham group; ^a^*p* < 0.05 vs. the Vag group; ^b^*p* < 0.05 vs. the HS group

The baseline values of HRV components did not show significant difference in all groups. In the In-Com phase of the HS group, LF and HF increased significantly. Furthermore, there was a slight increase in VLF. The increases of LF, HF and VLF were much pronounced in the De-Com phase and returned slowly back to their baselines in the Re-Com phase. The patterns of LF and HF during the De-Com and Re-Com phases in both HS and Vag + HS groups were similar, though the values in the Vag + HS group were lower than those in the HS group. Also, the increase in VLF in the De-Com and Re-Com phases of the Vag + HS group were lower than those in the HS group. The LF/HF ratio only increased at the end of Re-Com phase in the Vag + HS group (Fig. 3).

**Fig. 4:**
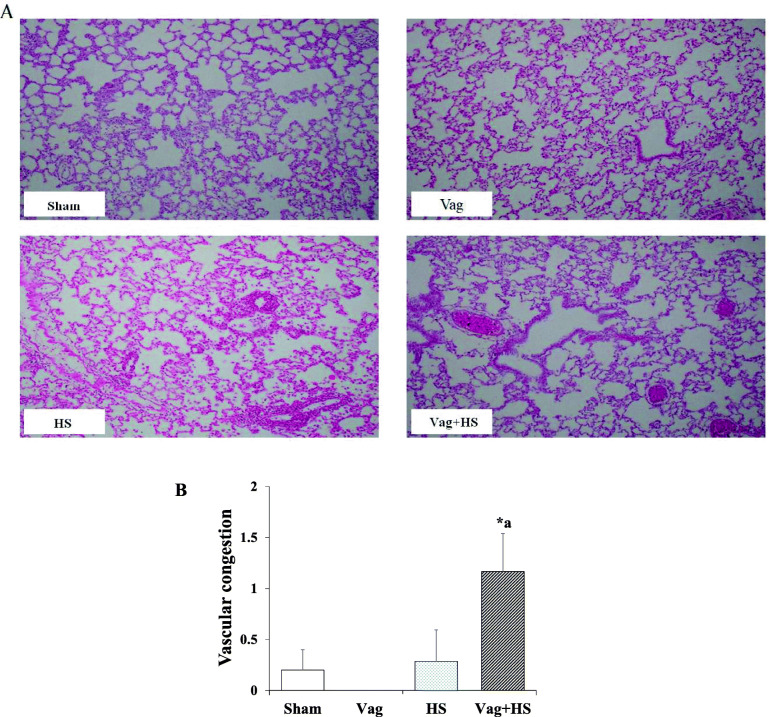
The hematoxylin-eosin staining with magnification of 100X (**a**) and vascular congestion (**b**) in the lungs of the Sham (*n* = 5), Vag (*n* = 5), HS (*n* = 7) and Vag + HS (*n* = 7) groups. Data are mean ± SE. ^#^*p* < 0.05 vs. the Sham group and ^a^*p* < 0.05 vs. the Vag group

Tables 1 and 2 indicate the arterial and venous blood parameters taken at the end of the steady-state period, and at the end of the experiments. There was no difference in all values at the baselines between groups. At the end of the experiments, the Hb concentrations in both HS and Vag + HS groups were lower than those in the Vag and Sham groups. However, it was only significant in the Vag + HS group compared with the Vag group. The PaO_2_/FIO_2_ ratio in the Vag + HS group was lower than that in the Sham group. Also, the decrease in PaO_2_ were along with the decrease in arterial oxygen content in Vag + HS group. The plasma lactate in the HS and Vag + HS group were insignificantly more than those in the Sham and Vag groups. (Table 3). Vascular congestion in the Vag + HS group was higher than those in the Sham and Vag groups (Fig. 4).

**Table 1:** The comparison of arterial blood gas parameters at baseline and at the end of the experiments

	Sham	Vag	HS	Vag + HS
**pH -1**	7.46 ± 0.009	7.46 ± 0.02	7.46 ± 0.015	7.47 ± 0.012
**PaCO**_**2**_**–1**(mm Hg)	34.78 ± 1.24	32.95 ± 0.89	32.14 ± 1.59	33.25 ± 0.65
**PaO**_**2**_**–1**(mm Hg)	68 ± 1.12	68.66 ± 3.84	62.66 ± 1.6	65 ± 2.12
**HCO**_**3**_^**−**^**-1**(mmol/L)	24.75 ± 0.50	23.55 ± 0.86	24.42 ± 0.73	24.4 ± 0.41
**BE-1**	1.2 ± 0.34	0.12 ± 1	1.56 ± 0.45	1.23 ± 0.59
**pH -2**	7.48 ± 0.007	7.48 ± 0.008	7.52 ± 0.013	7.51 ± 0.009
**PaCO**_**2**_**–2** (mm Hg)	30.17 ± 0.91	30.72 ± 1.28	26.46 ± 0.87	27.48 ± 1.19
**PaO**_**2**_**–2** (mm Hg)	73.24 ± 1.89	68.75 ± 1.25	67.83 ± 1.5	66 ± 1.82 ^*^
**HCO**_**3**_^**−**^**-2** (mmol/L)	23.07 ± 0.36	22.86 ± 0.65	22.68 ± 0.35	22.24 ± 0.68
BE-2	0.5 ± 0.24	0.12 ± 0.45	1.38 ± 0.42	0.51 ± 0.52

Data are mean ± SE in the Sham (*n* = 5), Vag (*n* = 5), HS (*n* = 7) and Vag + HS (*n* = 7) groups. * *P* < 0.05 vs. the Sham group; pH -1, PaCO_2_–1, PaO_2_–1, HCO_3_^−^-1 and base excess (BE-1) are the arterial blood parameters at baseline. pH -2, PaCO_2_–2, PaO_2_–2, HCO_3_^−^-2 and BE-2 are the arterial blood parameters at the end of the experiments. PaO_2_: arterial oxygen pressure, PaCO_2_: arterial carbon dioxide pressure. Barometric pressure: 630 mmHg

**Table 2:** The comparison of venous blood gas parameters at baseline and end of the experiments

	Sham	Vag	HS	Vag + HS
**pH -1**	7.43 ± 0.013	7.40 ± 0.02	7.43 ± 0.013	7.43 ± 0.009
**PvCO**_**2**_**–1**(mmHg)	40.8 ± 0.99	39.9 ± 0.62	36.58 ± 2.12	37.25 ± 1.4
**PvO**_**2**_**–1**(mmHg)	41.71 ± 0.64	42 ± 2	39.33 ± 1.8	40.4 ± 1.2
**HCO**_**3**_^**−**^**-1**(mmol/L)	26.85 ± 0.44	25 ± 1.42	25.08 ± 0.66	25.1 ± 0.58
**BE-1**	2.08 ± 0.32	0.06 ± 1.62	1.28 ± 0.32	0.93 ± 0.42
**pH -2**	7.43 ± 0.005	7.44 ± 0.009	7.45 ± 0.009	7.44 ± 0.014
**PvCO**_**2**_**–2**(mmHg)	37.92 ± 0.73	36.26 ± 0.87	34.85 ± 1.81	35.73 ± 1.32
**PvO**_**2**_**–2**(mmHg)	36.85 ± 1.45	36.25 ± 1.6	31 ± 2.16	27.34 ± 2.13^#*^
**HCO**_**3**_^**−**^**-2**(mmol/L)	25.77 ± 0.37	25.1 ± 0.48	24.9 ± 0.82	24.25 ± 0.61
BE-2	1.26 ± 0.18	1.16 ± 0.47	1.35 ± 0.51	0.7 ± 0.59

Data are mean ± SE in the Sham (*n* = 5), Vag (*n* = 5), HS (*n* = 7) and Vag + HS (*n* = 7) groups. * *P* < 0.01 vs. the Sham group; ^#^*p* < 0.05 vs. the Vag group. pH -1, PvCO_2_–1, PvO_2_–1, HCO_3_–1 and base excess (BE-1) are the venous blood parameters at baseline. pH -2, PvCO_2_–2, PvO_2_–2, And HCO_3_^−^-2 and BE-2 are the venous blood parameters at the end of the experiments. PvO_2_: venous oxygen pressure, PvCO_2_: venous carbon dioxide pressure. Barometric pressure: 630 mmHg

**Table 3:** The comparison between the hemoglobin and arterial oxygen pressure and contents in the experimental groups

	Sham	Vag	HS	Vag + HS
**Hb** (g/dl)	13.69 ± 0.72	14.7 ± 0.69	11.82 ± 0.53	11.19 ± 0.49 ^#^
**PaO**_**2**_**–2** (mmHg)	73.24 ± 1.89	68.75 ± 1.25	67.83 ± 1.5	66 ± 1.82 ^*^
**CaO**_**2**_**–2** (ml/100 ml blood)	17.82 ± 1.03	18.61 ± 0.82	14.98 ± 0.71	14.09 ± 0.53 ^#*^
**PvO**_**2**_**–2** (mmHg)	36.85 ± 1.45	36.25 ± 1.6	31 ± 2.16	27.34 ± 2.13 ^#*^
**CvO**_**2**_**–2** (ml/100 ml blood)	13.05 ± 0.68	13.95 ± 0.82	9.02 ± 0.82 ^#*^	7.06 ± 1.19 ^#*^
**PaO**_**2**_**-PvO**_**2**_**–2** (mmHg)	36.38 ± 2.78	32.5 ± 0.95	37.4 ± 3.78	39.9 ± 3.60
**CaO**_**2**_**-CvO**_**2**_**–2** (ml/100 ml blood)	4.76 ± 0.60	4.66 ± 0.55	5.95 ± 1.02	7.57 ± 0.66
**PaO**_**2**_**/FIO**_**2**_**ratio**	348.77 ± 9.01	327.38 ± 5.95	323.01 ± 7.41	314.76 ± 8.69 ^*^
Lactate (mmol/L)	13.65 ± 2.23	12.12 ± 2.44	23.48 ± 5.13	22.44 ± 3.94

Data are mean ± SE in the Sham (*n* = 5), Vag (*n* = 5), HS (*n* = 7) and Vag + HS (*n* = 7) groups. Hb: Hemoglobin concentration, PaO_2_–2: arterial oxygen pressure, PvO_2_–2: venous oxygen pressure, CaO_2_–2: arterial oxygen content and CvO_2_–2: venous oxygen content, PaO_2_-PvO_2_–2: arterial-venous oxygen pressure difference, CaO_2_-CvO_2_–2: arterial-venous oxygen content difference FIO_2_: fractional concentration of oxygen. *P < 0.05 vs. the Sham group; ^#^*p* < 0.05 vs. the Vag group. Barometric pressure: 630 mmHg. Normal PaO_2_/FIO_2_ ratio: 300 ± 10 mmHg

## Discussion

A few studies have reported the spectral analysis of HRV, and the role of subdiaphragmatic vagus nerve in class II hemorrhagic shock in conscious animals. Besides, the impact of subdiaphragmatic vagotomy on lung injury induced by hemorrhagic shock has not been illustrated yet [20]. In the present study, we indicated that the contribution of autonomic nervous system differs in three phases of class II hemorrhagic shock. The sympathetic activity in the In-Com phase shifts to the parasympathetic activity in the De-Com phase. Also, the parasympathetic activity is much pronounced in the Re-Com phase of hemorrhagic shock. There is an inverse relationship between the level of VLF and lung injury in the hemorrhagic shock.

No alteration was detected in MAP and HR at baseline and during the time course of experiments in the Sham and Vag groups, which suggests that the unilateral subdiaphragmatic vagotomy per se could not affect the hemodynamic and cardiac function.

The constant MAP in the In-Com phase of blood withdrawal in the HS group can be due to the increase in HR, which is supported by the results of the previous studies [2]. On the other hand, it has been indicated that the systemic vascular resistance, but not HR, increases markedly in conscious rats subjected to hemorrhagic shock [5]. However, in our study, we did not measure vascular resistance because of some limitations in the experimental preparations. Therefore, it can be suggested that at least a part of a constant blood pressure is linked to the increase in HR. There was not a significant variation in the parasympathetic component of HF [21] in the In-Com phase of HS group, though it tended to increase. However, a significant increase in LF may be due to the increase in sympathetic activity [22]. On the other hand, the LF/HF ratio, an indicator of sympathetic balance [23], did no change, which can be related to an insignificant increase in HF. Together, it is expected that the parasympathetic activity decreases and the sympathetic activity increases in this phase. In line with our results, Porter et al. showed that the increase of HR in the compensatory phase of hemorrhage, is mainly mediated by the increase in sympathetic activity, but not the parasympathetic drive [12]. By contrast, Troy et al. have reported that the constant heart rate in the initial phase of hemorrhagic shock is related to the decrease in parasympathetic drive, with no change in the sympathetic activity [2]. The difference in the experimental conditions may be influential in these dissimilarity results.

In our study, vagotomy did not alter the MAP in the In-Com phase of the Vag + HS group, whereas it increased HR. Therefore, the increase in HR may be linked to the decrease in parasympathetic activity, increase in sympathetic activity, or both.

The blood withdrawal resulted in a sudden drop in MAP and HR in the De-Com phase of the HS group similar to the previous studies [2]. HF increased in the De-Com phase of the HS group in agreement with other reports [12]. In addition, the contribution of the parasympathetic system to sudden cardiovascular collapse has been suggested in the splanchnic arterial occlusion method [24, 25]. Besides, an increase in LF was observed in the De-Com phase of HS in our study, causing the LF/HF ratio to remain unchanged. The increase in LF may be related to the increase in the sympathetic activity [9, 22]. However, there are some evidences, indicating that the LF band cannot be considered entirely as a sympathetic index [26]. To sum up, since the parasympathetic index of HF increases and HR decreases in the De-Com phase of HS, the dominant effect of the parasympathetic activity is suggested in this phase.

Although, the reduction in MAP in the De-Com phase of both HS and Vag + HS groups were similar, HR did not decrease in the Vag + HS group. However, the amount of blood withdrawn in this group was identical to that in the HS group. Therefore, it can be suggested that the main cause of pressure drop in this phase may be the decrease in vascular resistance, not reduced HR. We did not find a similar study indicating the effect of subdiaphragmatic vagotomy on HRV components during hemorrhagic shock. However, it has been suggested that subdiaphragmatic vagotomy may increase the plasma epinephrine following the disinhibition of the adrenal gland [8]. Consequently, it may prevent of bradycardia during the De-Com phase of hemorrhage in the Vag + HS group. On the other hand, HF and LF were high in the De-Com phase of the Vag + HS group, even though these parameters were lower than those in the HS group. Also, the LF/HF ratio was lower in the Vag + HS group. Taken together, the decrease in MAP, with no change in HR, concomitant with the results of HRV analyses suggest the less sympathetic activity compared with the parasympathetic activity in the De-Com phase of class II hemorrhagic shock.

In the Re-Com phase, the HR and MAP gradually returned back to the baseline in the HS group, and did not indicate a significant difference with the ones in the Sham group. All components of HRV were gradually recovered. A few studies have addressed the hemodynamic alterations and HRV in the Re-Com phase of hemorrhagic shock. In one study, the recovery was assessed within 30 min after hemorrhage [12], which was not enough time to return MAP and HR to their baselines. Our study was performed in a long time, so that the hemodynamic parameters returned to their baselines and being stable up to the end of the experiments. However, HR increased in the Re-Com phase of the Vag + HS group. There was no significant variation between HF during the time course of Re-Com phase in the Vag + HS group. As a result, the cause of increased HR cannot be directly linked to the decrease in the parasympathetic drive. In addition, the LF/HF ratio at the end of the experiments in the Vag + HS group was higher than that in the HS group, which may suggest a gradual increase in sympathetic drive in this time.

Our results showed a significant increase in VLF in the De-Com phase and at the onset of the Re-Com phase of the HS group. The increase in VLF in the Vag + HS group was lower than that in the HS group. These data suggest that the relationship between the HR and the level of VLF could be inversely proportional. The decrease in VLF is associated with the increase in HR. Numerous studies have suggested that the VLF band is a powerful indicator in predicting different diseases and injuries [27]. In our study, VLF in the Re-Com phase of the Vag + HS group was lower than that in the Sham group. Therefore, it can be argued that there is an inverse relationship between the level of VLF and organ dysfunctions including the lung in hemorrhagic shock.

The subdiaphragmatic vagotomy resulted in the decreases in PaO_2_ and PvO_2_ in the Vag + HS group. Also, the PaO_2_/ FIO_2_ ratio in the Vag + HS group was lower than that in the Sham group. Since the histological assessments indicated vascular congestion in the Vag + HS group, it can be proposed that the reduction of PaO_2_ and PaO_2_/FIO_2_ ratio in the Vag + HS group is linked to shunt or ventilation/perfusion mismatching in the lung [28]. In the hemorrhagic shock, the arterial-venous oxygen content difference increases due to the increased oxygen demand of the affected tissues. Therefore, the decrease in PvO_2_ in the Vag + HS group along with increased the arterial-venous oxygen content difference may be linked to the increase in tissue oxygen uptake by body organs including the heart in order to repay the oxygen debt [29]. Furthermore, the increase in plasma lactate was not significant in both HS and Vag + HS group which is confirming that lactate concentration can not use as an indicator for evaluating the patients with the class II hemorrhagic shock [30].

## Conclusion

In this study, we indicated the contribution of autonomic nervous system in three phases of class II hemorrhagic shock. The sympathetic system is predominant in the first phase, whereas the parasympathetic system is dominant in the second and third phases of hemorrhagic shock. Also, the subdiaphragmatic vagotomy may lead to lung injury in hemorrhagic shock. Taken together, the analyzing the HRV during different phases of hemorrhagic shock may pave the way for rapid diagnosis and treatment of the patients during critical condition in clinic.

## Data Availability

The data sets used and/or analyzed during the current study are available from the corresponding author on reasonable request.

## Abbreviations

HS: Hemorrhagic shock
Vag: Vagotomy
HRV: Heart rate variability
VLF: Very low frequency
LF: Low frequency
HF: High frequency
MAP: Mean arterial blood pressure
HR: Heart rate
Hb: Hemoglobin
PaO_2_: Arterial venous oxygen pressure
PvO_2_: Venous oxygen pressure
CaO_2_: Arterial oxygen content
CvO_2_: Venous oxygen content
PaO_2_-PvO_2_: Arterial-venous oxygen pressure difference
CaO_2_-CvO_2_: Arterial-venous oxygen content difference
FIO_2_: Fractional concentration of oxygen
